# Post-fire carbon and nitrogen accumulation and succession in Central Siberia

**DOI:** 10.1038/s41598-017-13039-2

**Published:** 2017-10-06

**Authors:** Markku Larjavaara, Frank Berninger, Marjo Palviainen, Anatoly Prokushkin, Tuomo Wallenius

**Affiliations:** 1 VITRI, Viikki Tropical Resources Institute, Department of Forest Sciences, PO Box 27, 00014 University of Helsinki, Helsinki, Finland; 2 Department of Silviculture and Biotechnology, Zhejiang A&F University, Lin’an, Zhejiang, China; 3 Department of Forest Sciences, PO Box 27, 00014 University of Helsinki, Helsinki, Finland; 4 V.N. Sukachev Institute of Forest SB RAS, Akademgorodok 50/28, Krasnoyarsk, 660036 Russia

## Abstract

Improved understanding of carbon (C) accumulation after a boreal fire enables more accurate quantification of the C implications caused by potential fire regime shifts. We coupled results from a fire history study with biomass and soil sampling in a remote and little-studied region that represents a vast area of boreal taiga. We used an inventory approach based on predefined plot locations, thus avoiding problems potentially causing bias related to the standard chronosequence approach. The disadvantage of our inventory approach is that more plots are needed to expose trends. Because of this we could not expose clear trends, despite laborious sampling. We found some support for increasing C and nitrogen (N) stored in living trees and dead wood with increasing time since the previous fire or time since the previous stand-replacing fire. Surprisingly, we did not gain support for the well-established paradigm on successional patterns, beginning with angiosperms and leading, if fires are absent, to dominance of *Picea*. Despite the lack of clear trends in our data, we encourage fire historians and ecosystem scientists to join forces and use even larger data sets to study C accumulation since fire in the complex Eurasian boreal landscapes.

## Introduction

Forest fires rapidly release carbon (C) stored in organic material, and may contribute to climate change by increasing atmospheric carbon dioxide (CO_2_). However, at a larger spatial and temporal scale, a pristine forest landscape can be assumed to be in a steady state, as forest patches recovering after fire sequester the same amount of C as is emitted during fires^1^. This balance is disturbed if fire severity or frequency is altered. These alterations could occur due to a changing climate or human-caused changes in ignitions or fire suppression. Fire frequency has been lengthening in the circumboreal forests during the past centuries^2,3^, and it appears unlikely that the rate of these changes would diminish in the upcoming decades. For example, current climate change driven by strengthening of the greenhouse effect is likely influencing fire regimes. If the decreasing trend is reversed, as is commonly believed to occur^4^, then ecosystem C density (C per unit land area) has less time to recover and mean C stocks are reducing in a given area.

Boreal forest fires influence climate change, not only by affecting ecosystem C by altering mean stand age, as described above, but also by floristically altering the vegetation^5^ and therefore impacting C accumulation, albedo^6,7^, volatile organic compound emissions^8^ and insulating snow depth impacting permafrost and related emissions^9^. For example, Siberian boreal forests are often divided into dark taiga, dominated by *Picea* and *Abies*, and light taiga, where *Larix* is the most important tree genus^10^. Frequent surface fires are believed to favour the development of light taiga with its fire-tolerant *Larix* and *Pinus sylvestris* trees. Mature individuals of these species have thick insulating bark in the lower parts of their trunks^11^. Several successive fires could therefore tip areas currently dominated by dark taiga into light taiga or to deciduous forest of trees, whose below-ground parts can survive fires and sprout rapidly (angiosperms). Reversely, the absence of fires could favour the shade-tolerant but fire-intolerant species (*Picea* and *Pinus sibirica*). These floristic shifts could influence the climate, not only by altering mean C stocks, but also via albedo, emissions of volatile organic compound and emissions triggered by melting permafrost.

Another mechanism for how fires influence climate change is through their impacts on nitrogen (N). Fires decrease N in ecosystems through oxidation and volatilization of N stored in biomass and surface soil^12–14^, which may have implications on primary production and C sequestration in N-limited boreal forests. Furthermore, fire-induced changes in soil abiotic environments (temperature, moisture, pH), microbial biomass and community composition, organic matter quality and vegetation alter N pools and fluxes during post-fire succession^15,16^.

Increased understanding of how fires influence the Siberian taiga and global climate is pivotal. Because of the large area and little direct human impact via logging or land conversion, even a small fire regime shift causing changes in these forests could drastically alter the global climate^17^ and influence the overall biosphere’s impact on global C balance^18^.

Vegetation succession and the post-fire accumulation of C and N can be studied experimentally^19^, but this methodology is not suitable for studying the recovery of boreal tree biomass, as the process takes centuries. Instead, the chronosequence approach has been the standard methodology, which assumes that the time since the last disturbance is the only difference between the studied plots^20^.

For example, in a chronosequence of plots with up to 151 years since a stand-replacing fire, *Picea mariana* –dominated “wet” sites accumulated some 86 g m^−2^ of ecosystem C annually, while dryer sites in Northern Manitoba, central Canada accumulated approximately half of that^21^. In a similar climate in central Alaska and the same dominant tree species, Mack *et al*.^22^ estimated significantly lower biomass accumulation rates of roughly 20 g m^−2^ of C annually in a mesic site and less in a dry site, using similar methodology. In a *Pinus sylvestris* -dominated landscape in central Siberia, C in the above-ground biomass accumulated in the more fertile sites at a rate of 100 g m^−2^ annually for 250 years, but at only 15% of this rate in the less fertile forest type^23^.

These chronosequence-based studies have provided precious information on C accumulation since fire occurrence. However, Johnson and Miyanishi^24^ raised their concern and claimed that the assumption that only time since disturbance causes the differences in vegetation is typically assumed without testing, and all four classic chronosequence studies that they examined exhibited other factors apart from time since disturbance that significantly influenced the vegetation. For example, Palviainen *et al*.^16^ showed that increasing anthropogenic N deposition was a major driver of the N balance in the northern boreal pine chronosequence. Perhaps more dangerously, limiting data analysis to the chronosequence approach, restricts ecologists to study edaphically and climatically uniform areas, which form only a small minority of e.g. most boreal regions. Furthermore, even in the uniform areas only those patterns matching the a priori perceptions of the researchers end up in chronosequence studies, as another pattern would incorrectly be perceived by these researchers as violating the uniformity criterion. A risk of circular reasoning therefore exists, and chronosequence studies are likely to reinforce the prior views of the researchers, but are unlikely to give unexpected results. E.g. when floristic succession is studied, changes that are in conflict with the prior understanding might be wrongly perceived as caused e.g. by soil variables that are difficult to study and the researcher might move to an area matching the classic thinking. These problems with the chronosequence approach are especially significant in Eurasian boreal forests, which typically have non-stand-replacing fires^25^. Using a single variable to describe the impact of past fires in such circumstances can be misleading.

Our objective was to quantify C and N accumulation and changes in tree species composition and stand density since fire occurrence in central Siberia based on an inventory-style sampling in which we chose plot locations prior to visiting the area. This approach is immune to the problems of the chronosequence approach described above, but is challenging for other reasons. The acceptance of plots varying edaphically or climatically or where the severity of previous fires has varied causes variation in the results, and only a large data set is likely to reveal clear trends. Secondly, fire history is challenging to study in a complex area compared to a simple “one species and time since stand-replacing fire -landscape”, and could potentially be influenced by edaphic or climatic conditions that could confuse the interpretation of the results.

To better take the variability of fire severity into consideration, we related vegetation succession and C and N accumulation not only to the time since previous fire occurrence, but also to forest age, which can be considered a proxy for the time since the previous stand-replacing fire, but could also be determined by another stand-replacing disturbance or the maximum age of the tree species. We hypothesized that:

1. C and N in living trees increase with increasing forest age and time since fire because trees are killed in fires.
2. C and N in the understorey and moss layer and humus layer increase with increasing forest age and time since fire occurrence because they burn partly.
3. C and N in snags and fallen logs decrease with increasing forest age and time since fire occurrence because trees are killed by the fire, but increase when more time has passed because of increasing tree mortality^26^.
4. The decrease of C and N in snags and fallen logs during the first decades is less significant than the increase in other pools and therefore overall ecosystem C and N increase with forest age and time since fire occurrence.
5. The biomass proportion of sprouting fire-intolerant tree species decreases with increasing forest age, as they are able to grow rapidly after stand-replacing disturbance^27^.
6. The biomass proportion of non-sprouting fire-intolerant tree species increases with increasing forest age and time since fire, as their mortality is high in fires and they need a long time to recover^27^.
7. The biomass proportion of non-sprouting fire-tolerant tree species decreases with increasing time since fire, as their mortality in fires is lower than that of the other groups^10^.
8. Stand density (number of tree individuals per unit area) decreases with increasing forest age and time since fire after the first decades^27^, as self-thinning is likely to outweigh the opposite impact of more time granted for reproduction and regrowth.

Hypotheses 1, 2 and 4 were directly based on fire chemistry while we defined the rest of them based on studies conducted elsewhere.

## Results

We measured a total of 943 living trees, 146 snags and 313 fallen logs. The living trees belonged to nine tree species (genus *Salix* is counted as one species). Only *Larix* occurred on all 46 plots, and the majority of species occurred on fewer than 10 plots (Table 1). The thickest *Larix* and *Pinus sylvestris* individuals had a dbh of over 50 cm, but only *Larix* surpassed 30 m in height. *Larix* contributed 72.5% of the total living tree biomass, followed by *Pinus sylvestris*, *Betula* and *Picea*. The remaining five species each contributed less than 1%. The basal area averaged 17.9 m^2^ and ranged from 1.1 m^2^ to 46.9 m^2^ on the plots. The above-ground biomass averaged 8.5 kg m^−2^ and its C content averaged 4.3 kg m^−2^ (from 0.2 kg m^−2^ to 14.6 kg m^−2^). The C content of snags averaged 0.9 kg m^−2^ (from 0.0 kg m^−2^ to 4.3 kg m^−2^), while that of fallen logs averaged 1.9 kg m^−2^ (from 0.0 kg m^−2^ to 6.9 kg m^−2^), that of the understorey and moss layer averaged 1.2 kg m^−2^ (from 0.3 kg m^−2^ to 5.0 kg m^−2^) and finally that of the humus layer averaged 1.7 kg m^−2^ (from 0.3 kg m^−2^ to 13.9 kg m^−2^). The humus layer thickness ranged from 0 cm to 27 cm with an average of 4.4 cm, and the moss layer from 0 to 26 cm with an average of 5.3 cm.

**Table 1:** Information on the nine species, with heights of at least 1.9 m, found on the plots. These are referred to only by their genus name in the main text, except for the two species of *Pinus*.

**Species**	**Group**	**Occurrence (of 46 plots)**, ***Larix*** **occurred on all plots**	**Maximum dbh (cm)**	**Maximum height (m)**	**Mean number of stems in all plots (ha** ^**−1**^ **)**	**Biomass (%)**
*Alnus viridis*	Fire-intolerant sprouting	7	6	7.1	531	0.6
*Betula pubescens*	Fire-intolerant sprouting	36	21	24.0	2802	7.6
*Larix gmelinii*	Fire-tolerant non-sprouting	46	52	31.3	1741	72.5
*Picea obovata*	Fire-intolerant non-sprouting	15	24	17.3	356	4.6
*Pinus sibirica*	Fire-intolerant non-sprouting	3	13	12.1	17	0.2
*Pinus sylvestris*	Fire-tolerant non-sprouting	24	52	27.6	815	14.1
*Populus tremula*	Fire-intolerant sprouting	5	25	24.2	68	0.4
*Salix spp*.	Fire-intolerant sprouting	7	3	5.5	233	0.1
*Sorbus sibirica*	Fire-intolerant sprouting	2	2	3.9	44	0.0

**Figure 1:**
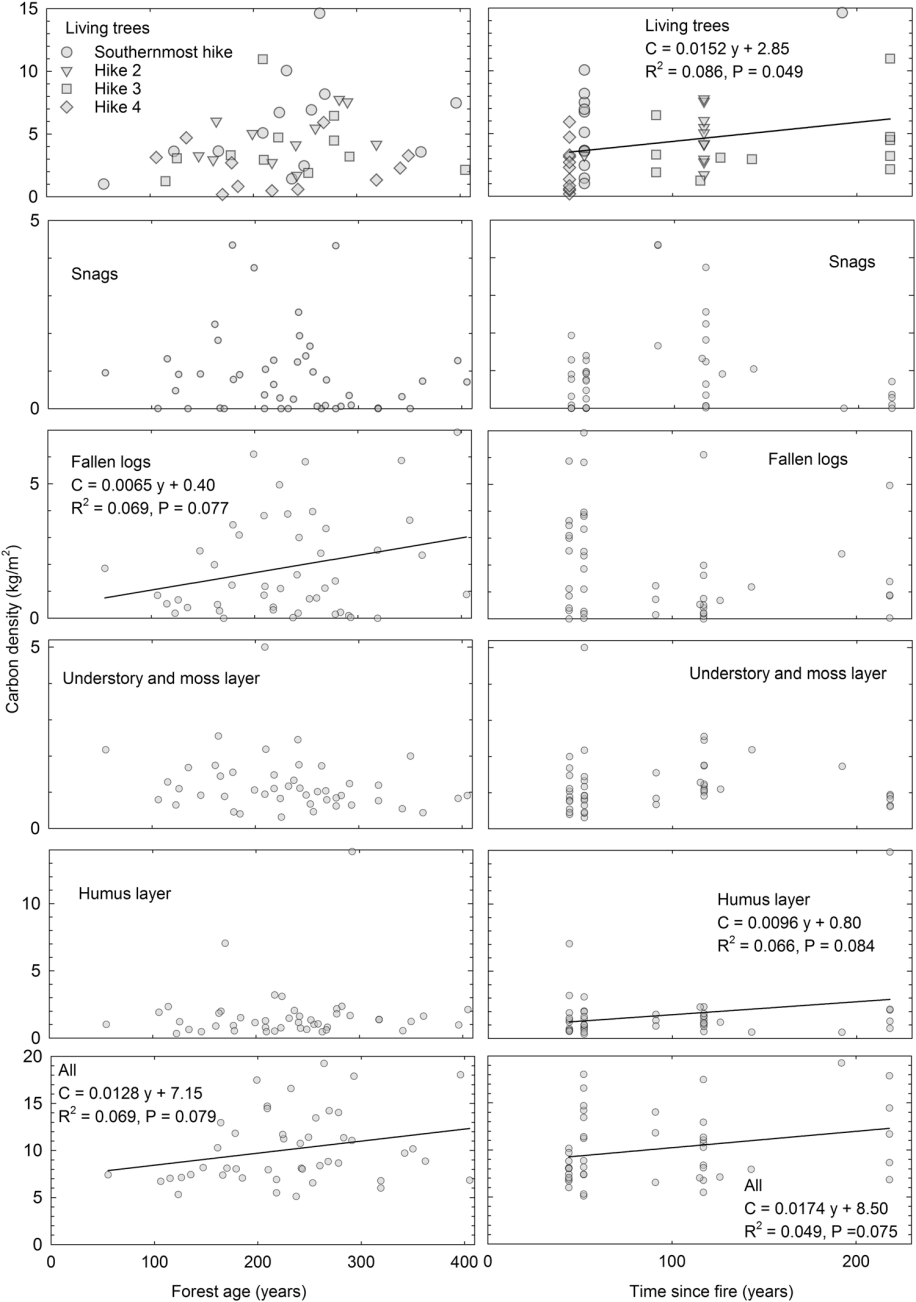
C density relative to forest age and time since previous fire occurrence. The top two panels show only plots sampled during same hikes with same symbols.

Despite our demanding sampling, the data did not reveal statistically significant trends in most of the studied relationships. When 0.05 was used as the P-value, increasing C content in living trees with increasing time since fire was the only statistically significant trend (Fig. 1). When the P-value limit is relaxed to 0.1, four other rising trends became statistically significant.

The mineral soils under the humus layer were mainly clays and silts. The moss layer was dominated chiefly by *Pleurozium*, *Cladonia*, *Hylocomium*, *Dicranum* and *Sphagnum*. *Vaccinium* was by far the most dominant genera in the grass and draft shrub layer, accompanied by *Carex*, *Rhododendron*, *Linnaea*, *Salix*, *Epilobium* and the family Poaceae. The shrub layer consisted mainly of small trees, but individuals belonging to genera *Rosa* and *Juniperus* were additionally present. We tried to understand the causes of the scatter in Fig. 1 by taking into account the texture of mineral soil or coverage of understory vegetation based forest types or fertility classes but these additional variables did not help in interpreting the data.

**Figure 2:**
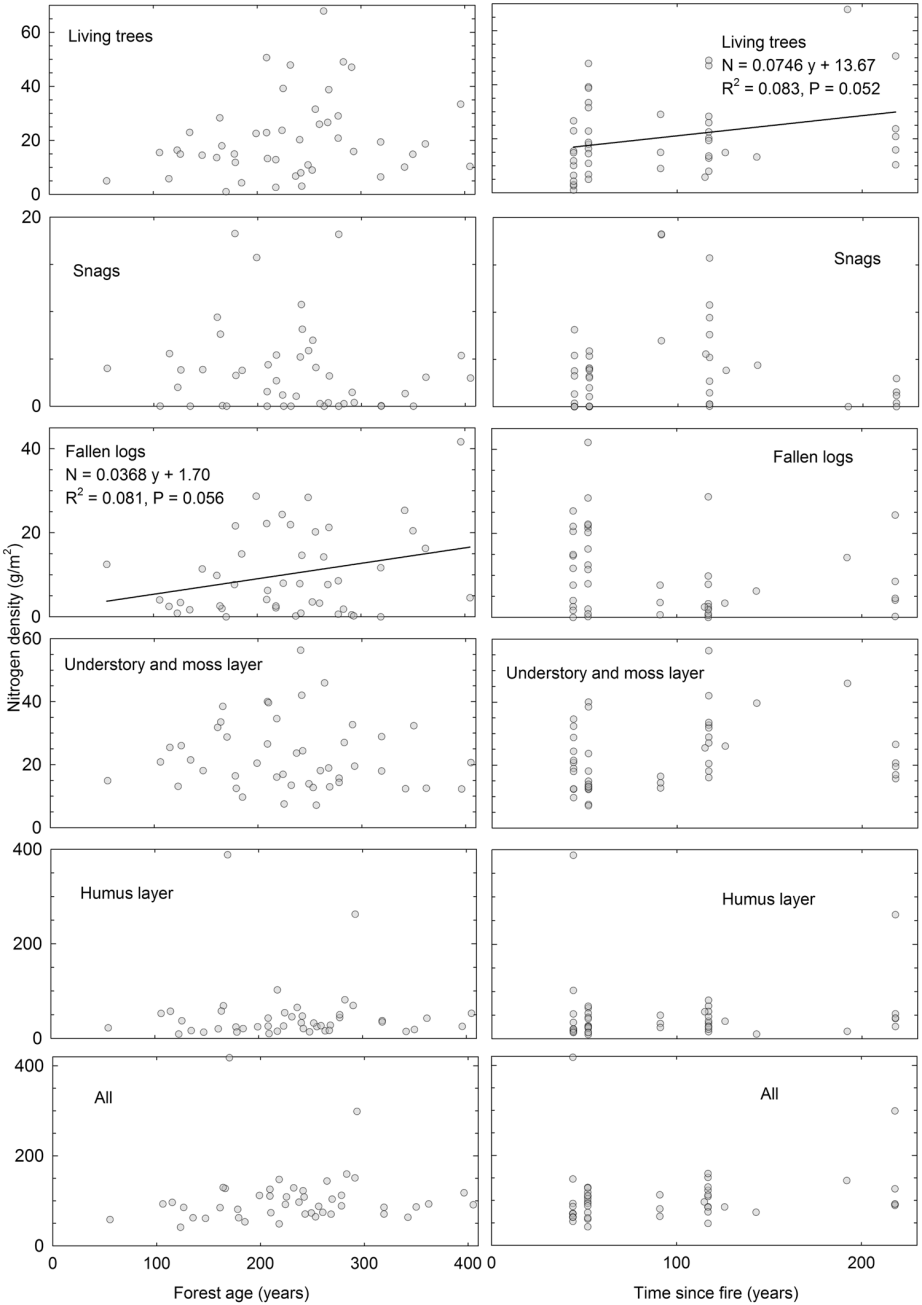
N density relative to forest age and time since previous fire occurrence.

The N pool in living trees increased significantly since fire, while that of fallen logs increased with forest age (Fig. 2). A large proportion of N was stored in the humus layer. In areas where the time since fire was ≤52 years, the organic layer N pools accounted for 35% of the total ecosystem N and in the plots with longest time since fire (218 years), this proportion was as high as 51%.

**Figure 3:**
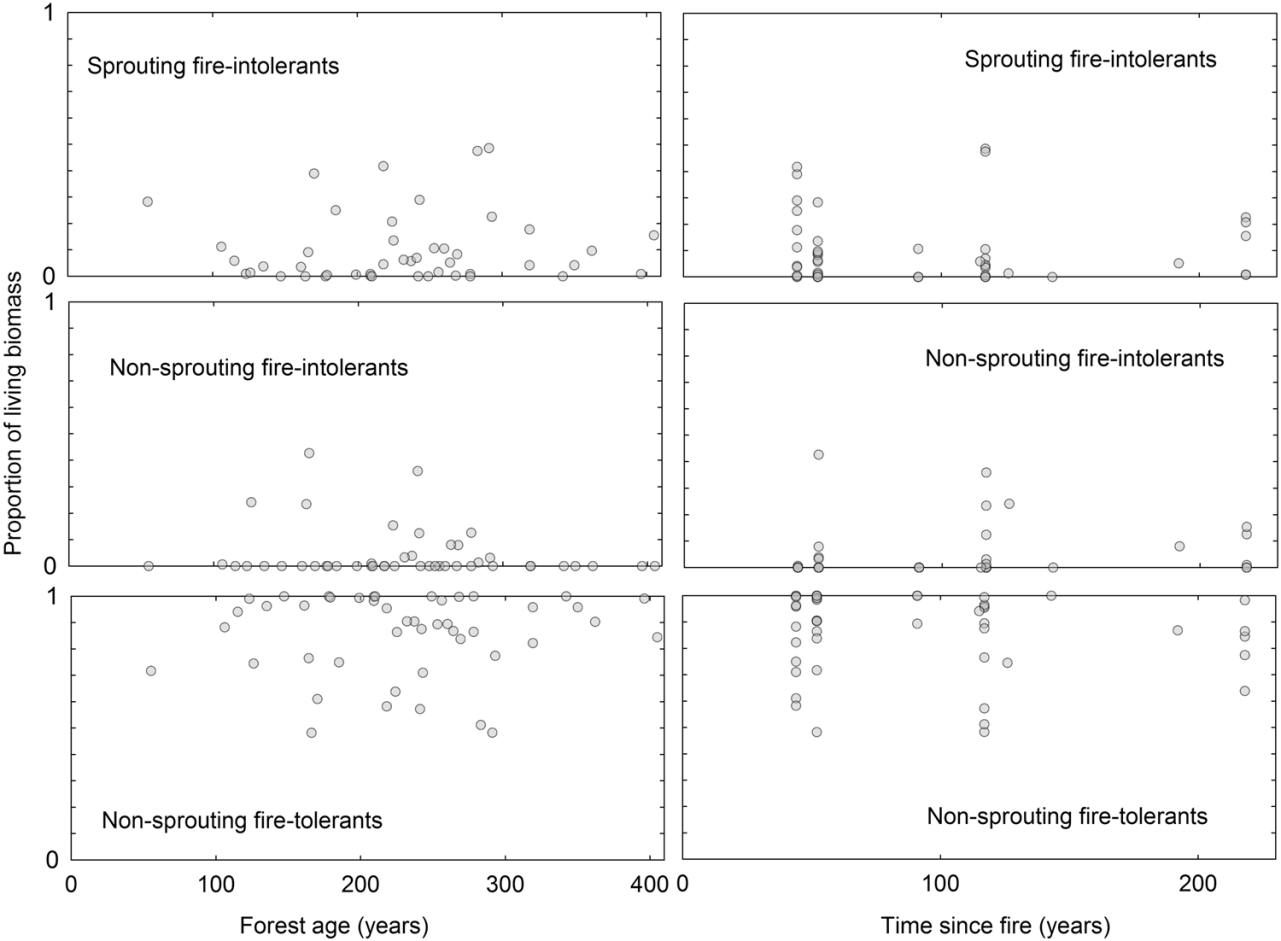
Biomass proportion of tree functional groups relative to forest age and time since previous fire occurrence.

Our plots on floristic tree succession showed even less trends (Fig. 3). Sprouting fire-intolerants, i.e. the angiosperms, had a small biomass share that never exceeded 50%, but this was not influenced by forest age or time since fire occurrence. The pattern is very similar for non-sprouting fire-intolerants, *Picea* and *Pinus sibirica*, and therefore naturally also the proportion of the remaining group of non-sprouting fire-tolerants *Larix* and *Pinus sylvestris* did not reveal trends.

**Figure 4:**
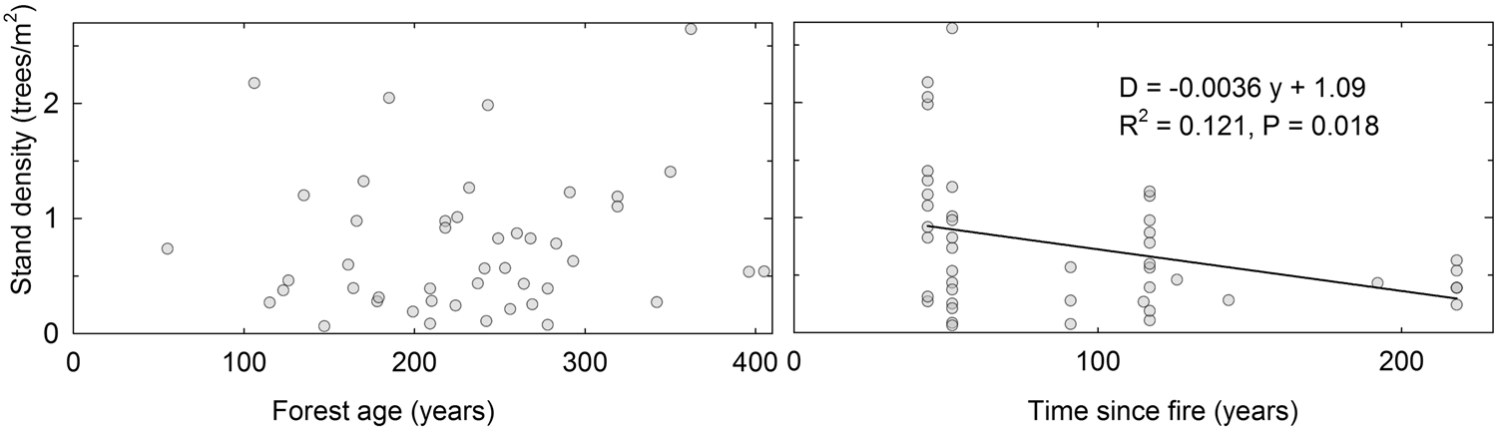
Number of trees of at least 1.9 m in height per unit area relative to forest age and time since previous fire occurrence.

Stand density was not influenced by forest age (Fig. 4). However, the decreasing trend of lower stand density with increasing time since fire was statistically more significant than any of the trends related to the main focus of this paper.

In addition to explaining C, N, floristic composition of trees and stand density with forest age and time since fire, we conducted some modelling considering all dated fires for each plot on C in living trees. However, even this failed to explain the large scatter (Supplementary Info File).

## Discussion

We understood prior to the fieldwork that our inventory approach, which is more objective compared to the standard chronosequence method, requires sufficient numbers of plots to reveal any trends. However, we were surprised that our study area was so heterogeneous that even with our 46 plots we were unable to expose many statistically significant trends. Our hypotheses 1, 2 and especially 4 were directly derived from the fact that burning releases C and N that have to be recovered back into the ecosystem. However, we found only weak support for these hypotheses (Figs 1 and 2). In the case of dead wood (hypothesis 3) we expected the legacy of dead wood produced by the fire to first dominate, and the trends for snags and fallen logs to initially decrease for the first decades and then be reversed. As each of our plots had not experienced a fire in at least 44 years, a rising trend should have been visible, but this was not the case.

Our weak trends hint of annual C accumulation of approximately 15 g m^−2^ for living trees and 17 g m^−2^ for all ecosystem C above the mineral soil since fire occurrence. These number are small compared to the up to 100 g m^−2^ of C that accumulates annually in a similar but somewhat warmer climate in Canada^21^. However, because of severe stand-replacing fires in North America the situation is completely different from our area, which is dominated by less severe surface fires that do not release so much resources for the new tree cohort and therefore the stand reaches its self-thinning or “stagnative equilibrium”^28^ stage more rapidly. It is also possible that many chronosequence studies have been conducted in areas with clearer and more rapid succession than on average, therefore biasing the overall picture. Furthermore, our data set did not include plots with less than 44 years since fire occurrence. It is likely that these first decades have faster C accumulation thanks to warming of the soil and withdrawal of the permafrost, resulting in increased nutrient availability^29^.

In addition to time since fire, we plotted C densities also relative to forest age, i.e. the age of the oldest looking tree on each the plot. We did this to gain an idea of C and N accumulation based on two thresholds in the fire severity gradient ranging from fires undetectable with fire history methods^30^ to stand-replacing fires. Again, the weak trends cause vague discussions on the differences between C accumulation with increasing forest age and time since fire occurrence, but the accumulation of all ecosystem C is surprisingly similar (bottom left and right in Fig. 1). With a larger data set it is likely that the higher starting C density at the beginning of succession when all detectable fires were included (bottom right in Fig. 1) would become evident.

Post-fire annual N accumulation rate was 0.07 g m^−2^ for living trees (Fig. 2). The weak trend (P = 0.25) for N accumulation above the mineral soil was 0.19 g m^−2^. Boreal forests have been reported to annually accumulate 0.29–0.33 g N m^−2^ ^31,32^. Our post-fire N accumulation rate was lower than this range, but quite the same order of magnitude as the values (0.21–0.23 g N m^−2^ yr^−1^) reported for sub-arctic *Pinus sylvestris* forests after non-stand-replacing fires^16^. Previous studies have reported either decreases or no change in soil N pools after forest fires^1,12,13,15,16^. We found that unlike for C, a large proportion of ecosystem N pools were stored in the humus layer (Fig. 2).

We expected clear trends related to groups of tree species defined based on fire tolerance and sprouting ability. We expected these not only because of mechanisms described in the list of hypotheses (5, 6 and 7), but also due to positive feedbacks. E.g. fire-intolerant *Picea* not only benefits from the absence of fires, but it also shades and keeps the understorey more humid than under light-demanding species and therefore lowers the likelihood of fire. *Larix* and *Pinus sylvestris* contrastingly benefit from fires killing other species and reducing competition and at the same time allowing light to dry the mosses, lichens and litter and increasing the likelihood of fire. Such positive feedbacks could cause relatively sudden changes, where one forest type shifts to another as a result of climate or fire regime changes. These change are unlikely to be reversed even if climate- or fire-related drivers revert to their previous state. Similarly, these positive feedbacks could cause spatially sudden jumps from dark taiga to light taiga, as is the case in central Siberia^10^. Surprisingly, our data did not reveal any trends related to tree species biomass proportions when plotted against forest age or time since fire occurrence (Fig. 3). This could be because the disturbance return interval was so short that early successional species were still abundant in all the plots, or perhaps the legacy of fire history over five hundred years could still be determining the current species distribution. It is also possible that our data set was just too small to show the supposed dark taiga succession of late successional *Picea* replacing *Betula* with increasing time since disturbance^27^.

Despite our trends in C accumulation being weak, they can be used with caution to speculate on the C implications of changing fire regimes due to direct human impacts on ignitions or fire spread via firefighting. First assume that the intercept, 8.50 kg m^−2^ of the regression in bottom right of Fig. 1, is the density of ecosystem C (above the mineral soil) immediately after a fire and that C increases at an annual rate of 17.4 g m^−2^. Then the lengthening of the fire cycle from 52 years in the 18^th^ century to 164 years in the 20^th^ century^3^ would translate into an increase of 0.97 kg m^−2^ assuming that nothing else changes and in an increase in C emission per fire from 905 g m^−2^ to 2,854 g m^−2^, which are low compared to fuel consumption levels gained mainly from experimental burnings reviewed by Kukavskaya *et al*.^33^. When C implications of the longer fire cycle in the study area are extrapolated to the entire Russian forest area^34^, such a change would translate in an increase of 7.9 Pg of C in two centuries, which is close to the recent annual global anthropogenic emissions^35^. When divided over two centuries, the annual sequestration of 0.04 Pg of C is very small compared to the increased biomass based on remote sensing^36^. However, our estimate is likely to be conservative, as the independence assumption of the departure point of C accumulation likely causes an underestimation in the C implications of an altered fire regime. In reality, C density prior to the fire probably positively influences the density after fire, i.e. repeated frequent fires could pull the post-fire ecosystem C density downwards fire after fire. The changes in tree species composition triggered by changes in fire regime, as discussed in the two previous paragraphs, could also have significant impacts in either direction.

Similarly, C impacts of the predicted shortening fire cycle due to climate change^37^ can be estimated, despite these speculations being even less certain. Firstly, because climate and fire danger simulations are uncertain – even knowing whether fires are increasing or decreasing, assuming no change in fire suppression, is an open question. Secondly, because of a warming climate, CO_2_ fertilization and N deposition are directly speeding up C accumulation in central Siberia^38^.

With our methodology we were able to avoid certain problems related to the chronosequence approach, but we faced some new methodological challenges. We did not concentrate our sampling on a uniform homogenous area, but still had to exclude some land ecosystem types, i.e. peatlands. If the thickness of the humus layer increases with time since fire occurrence^39^, and this depth is used to define a peatland, then inevitably if fire is absent for long enough a site is classified as peatland and is excluded from the sampling, which could truncate our data set and even cause bias. However, it is unlikely that this causes significant problems in our study despite two plots included in the sampling were clearly paludified and had thicker humus layers with much higher C and especially N density than all the others (outliers in the humus layer in Figs 1 and 2). Another complication is caused by our underlying assumption that edaphic or climatic heterogeneity do not influence the fire regime within our area, which certainly is far from the truth in intensively human influenced boreal Europe during the 18^th^ and 19^th^ centuries^40^. Fortunately, the large size of fires^3^ relative to the spatial pattern of edaphic heterogeneity in our study area mitigates the danger of bias caused e.g. by more common fires in dryer and more productive soils, which would lead to an underestimation of C recovery.

The research communities focusing on soil C and fire history have traditionally been distinct in boreal Eurasia and only a few soil C studies even mention forest fires despite most of Eurasian boreal forests having burned in the past centuries^41^ and the humus layer is likely still recovering rapidly from the previous fire, as this can take millennia^42^. Because of the separation of these two research communities, past fires are not typically discussed when past^43^ or future^44^ soil C has been modelled. We hope that our paper coupling a meticulously implemented fire history study^3^ with ecosystem C sampling, encourages more similar studies. Hopefully these will incorporate even more plots in Eurasian boreal forests and elsewhere, which would lead to a better understanding of past and future ecosystem C as fire regimes have changed and will continue to change.

## Methods

**Figure 5:**
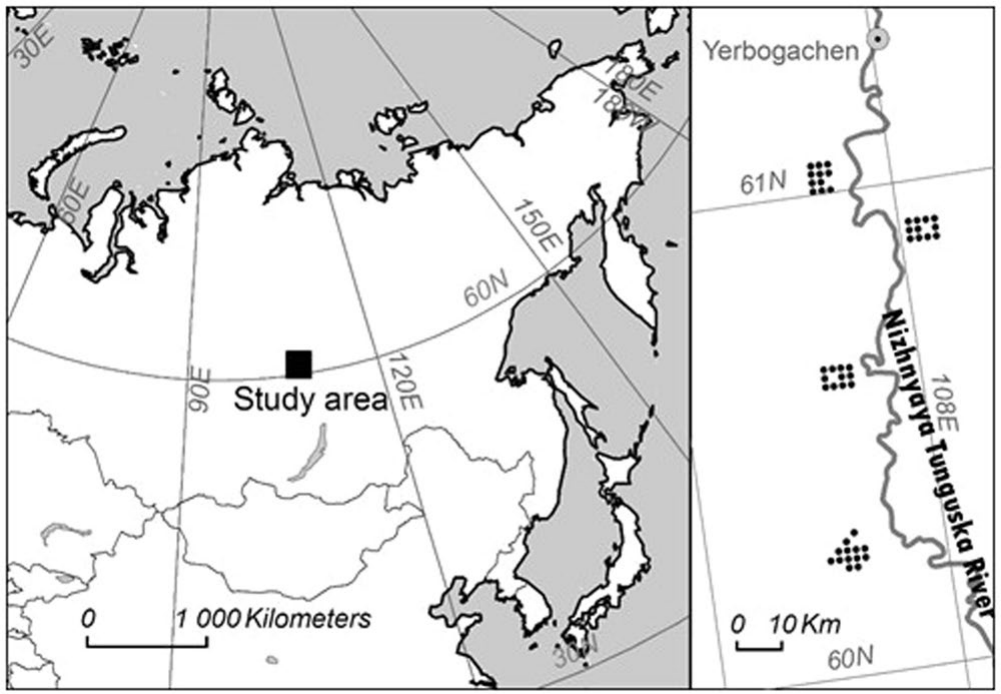
Map showing the location of the study area in eastern Russia (left) and the 46 plots on both sides of the river Nizhnyaya Tunguska (right). The map was modified from^3^.

We wished to accomplish the research objectives by studying a remote and sparsely populated part of the Siberian taiga, which nevertheless represents a vast area of the little-studied region. We chose “middle taiga”^45^ *Larix-*dominated forests along Nizhnyaya Tunguska, a tributary of the Yenisei River, in northern Irkutsk Oblast close to the town of Yerbogachen, where inhabitants are concentrated in villages along the river (Fig. 5). The river is also the principal mean of transport in the area. However, with the exception of spring floods, the rapids of the river make it impossible for larger boats to travel on it.

The area has an ultra-continental climate. Yerbogachen has a mean temperature of 16.6 °C in July, −31.8 °C in January and annual precipitation of 401 mm, 69% of which falls during the wetter six-month-period from May to October^46^. The area has discontinuous permafrost^47^.

We carried out the fieldwork in August and September 2003 by completing four six-day hikes as far inland as possible to minimize the impact of the river (Fig. 5). The vicinity of the river is likely to be more influenced by humans and the fire regime could be impacted by the river acting as a natural fire break or because of the spring floods, unusual soils and vegetation in the vicinity of the river. The elevation of our plots ranged from 280 m to 440 m above sea level with higher elevations in the South reducing the climatic variability within our study area. The local topography was dominated by gentle slopes rarely more than 50 m high. We overoptimistically aimed to inventory a higher number of plots during the first and southernmost of the hikes, but had to settle for fourteen plots. During the remaining three hikes we compromised and completed twelve plots during each hike, resulting in a total of fifty plots. However, we did not collect data from plots on peatland, defined as having a humus layer of 30 cm or more. We encountered one peatland plot during each hike, and therefore collected data from 46 plots. We used a rough map to choose the locations of all the hikes and the plots prior to the field trip. The map contained very little information other than the main river and we positioned the hikes on outer sides on a twist of the meandering river to avoid having plots surrounded by the river from three sides. The hikes were some thirty kilometers apart along the river, and the plots were located two kilometers from the neighbouring plot or plots. We encountered signs of human activity, such as isolated cabins for sable hunters, during three out of the four hikes, but none were close to the plots and we saw no signs of significant logging anywhere. Photos from the plots from the southernmost and northernmost hikes are available in the Supplementary Info File.

The sampling was designed to serve both a published fire history study^3^ and this paper, which is partly based on results from the study. In summary, the fire history reconstruction was based on samples taken from trees with fire scars and from the oldest-looking trees on the plots with a 100 m radius. The scars analysed with dendrochronological techniques revealed the year of the latest fire and the age of the oldest-looking tree revealed the forest age. GPS coordinates of the plot centres were predefined before starting the hikes. To prevent underrepresentation of the densest thickets that are naturally avoided while walking and searching for the set GPS point, we centred the plots 10 m from the GPS point.

We measured living trees that were at least 1.9 m tall. Trees with a dbh (diameter at 1.3 m height) of at least 20 cm (rounded to the closest cm) were included from a plot with a radius of 10 m, thinner trees but with a dbh of at least 10 cm were included from a plot with a radius of 5 m and even thinner trees from a plot with a radius of 2.5 m. In addition to measuring dbh with a calliper, we measured height with a Vertex (Haglöf Sweden AB, Långsele, Sweden) that is based on the tangent method^48^. We estimated the height of the shortest trees based on their height relative to the known height of the fieldworkers.

We measured snags (dead standing trees) as living trees with one exception. When a snag had a broken top we recorded a rough visual estimate of the top diameter of the remaining snag.

Fallen logs, or pieces of coarse wood debris, were included in the sampling when their thicker end was at least 10 cm in width and located less than 10 m from the plot centre. We measured the length of each fallen log and the diameter at both ends. When the shape differed from that of a cut cone, we recorded the diameters that we visually estimated, to calculate the true volume of the fallen log when volume is computed assuming the shape of a cut cone. We additionally performed a simplified version of the “knife test”, in which we pressed a knife vigorously into the wood, and recorded its penetration level^49^. We used the following classes: 1) blade does not penetrate significantly, 2) blade penetrates, but not all the way and 3) blade penetrates fully.

Due to the challenging logistics, we could not transport heavy soil samples to a laboratory. We measured the thickness of both humus and moss layers at seven locations along the twenty-meter axis running through the plot. In addtion we conducted light destructive soil sampling. From each plot, with the help of a 7 cm square plastic plate, we cut samples with a knife from two opposite points 4 m from the plot centre. We collected the understorey vegetation and moss layer into one plastic bag and the humus or organic layer down to the mineral soil into another bag. The understorey vegetation and moss layer included all living woody plants less than 1.9 m in height, non-woody plants and litter, including fine woody debris and branches of fallen logs. The humus layer included all materials, including living roots, below the lower parts of the living mosses and lichens and above the mineral soil, in which more than half of the volume is composed of mineral particles. We dried, weather permitting, these bagged samples in the field to avoid decomposition during the transportation lasting over two months at maximum.

In addition we classified the mineral soils under the humus layer based on hand identification. We also identified the floristic composition of dominant plant genera at three layers.

We transported both the fire history, and samples from the understorey and moss layer and the humus layer to the University of Helsinki, Finland. In the laboratory, soil samples were dried (60 °C, 24 h), sieved through a 2 mm sieve, and ground. The C and N concentrations of the samples were determined with a CN-analyzer (Leco CSN-1000, Leco Corp., St Joseph, MI, USA) and subsamples were taken for dry mass determination (105 °C, 24 h). All data is available in Supplementary Dataset File.

We estimated the C content of living trees based on our measured heights and dbh’s and the allometric equations by assuming that 50% of the living biomass was C, which is likely an accurate value for our region^50^. Nitrogen concentrations of living trees were obtained from the literature^51–55^. Because of the importance of *Larix* in our plots, we developed new allometric equations for it based on the destructive sampling of 112 *Larix* trees close to the Tura research station some 500 km from our main study area (Supplementary Info File). For the second most important species, *Pinus sylvestris*, we used published equations from approximately 1,000 km west of our study area^23^. Some of their equations were age-based. We used the equations for 95-year-old trees for our trees with a dbh of 0.1 m or less, and the equations parameterized for age classes of 138, 200, 204 and 215 years for trees thicker than that. Central Siberian allometric equations were not available for the remaining tree species. We computed the biomass of *Pinus sibirica* trees with the same equations as for *Pinus sylvestris* trees, but multiplied with the wood density ratio, 340 kg m^−3^/380 kg m^−3^, given by Harmon *et al*.^56^. For the remaining species we unfortunately had to use equations parameterized for North European trees, but based on our visual field comparisons large biases were unlikely. We estimated *Picea* ^57^ and *Betula* ^58^ biomasses directly, but performed similar wood density -based calibration as for *Pinus sibirica* for the remaining rare angiosperm species or genus *Salix* (which is listed in Table 1 as the ninth “species” for simplicity). We used the *Betula* equation modified by the ratio of mean wood density in a wood density database and *Betula* wood density from Finland (475 kg m^−3^)^59^ as for *Pinus sibirica*. We used a wood density of 350 kg m^−3^ for *Populus* ^56^, while the densities for *Alnus* (417 kg m^−3)^ *, Salix* (408 kg m^−3)^ and *Sorbus* (573 kg m^−3^) were based on the means of all the Asian records in the database^60^.

As probably even a larger share of snags than living trees are *Larix* due to their durable wood, we computed snag necromasses using the same equations as for living *Larix* trees. However, we assumed a decrease in wood density and change in the proportion of C due to decomposition. We used reported values^61^ for *Pinus sylvestris*, the second most important species in our plots because of the similarity between it and *Larix* wood, and their knife test class 1, i.e. a decrease of 9.1% in C. For snags with tops missing we assumed constant tapering based on the top and 1.3 m height diameters, a shape of a cut cone and wood density of 460 kg m^−3^ ^56^, but with the same decrease of 9.1% in C.

As with snags, we assumed all fallen logs to be *Larix* wood, and computed their volume assuming a shape of cut cones. To take into account the decrease of C due to decomposition, we used the data reported for *Pinus sylvestris* by combining the knife test classes 1 and 2 of Mäkinen *et al*.^61^ to correspond to our most intact class, their class 3 to correspond to our intermediate class and their classes 4 and 5 to correspond to our most decayed class. The assumed decreases in C were 21.2%, 45.4% and 77.1%, respectively, and are mainly caused by a decrease in wood density with only minuscule impact from changing proportion of C^61^. Based on previous studies, N concentrations were assumed to be 0.21%, 0.27% and 0.41% in decay classes 1, 2 and 3, respectively^62,63^.

## Electronic supplementary material

Supplementary Info File

Supplementary dataset file

## References

[1] Wirth C (2002). Fire and site type effects on the long-term carbon and nitrogen balance in pristine Siberian Scots pine forests. Plant and Soil.

[2] Wallenius T (2011). Major decline in fires in coniferous forests–reconstructing the phenomenon and seeking for the cause. Silva Fennica.

[3] Wallenius T, Larjavaara M, Heikkinen J, Shibistova O (2011). Declining fires in Larix-dominated forests in northern Irkutsk district. International Journal of Wildland Fire.

[4] de Groot WJ, Flannigan MD, Cantin AS (2013). Climate change impacts on future boreal fire regimes. Forest Ecology and Management.

[5] Shuman JK (2017). Fire disturbance and climate change: implications for Russian forests. Environmental Research Letters.

[6] Randerson JT (2006). The impact of boreal forest fire on climate warming. science.

[7] Landry JS, Matthews HD (2016). Non-deforestation fire vs. fossil fuel combustion: the source of CO2 emissions affects the global carbon cycle and climate responses. Biogeosciences.

[8] Unger N (2014). On the role of plant volatiles in anthropogenic global climate change. Geophysical Research Letters.

[9] Romanovsky V (2010). Thermal state of permafrost in Russia. Permafrost and Periglacial Processes.

[10] Schulze ED (2012). Factors promoting larch dominance in central Siberia: fire versus growth performance and implications for carbon dynamics at the boundary of evergreen and deciduous conifers. Biogeosciences.

[11] Shorohova E, Kneeshaw D, Kuuluvainen T, Gauthier S (2011). Variability and dynamics of old-growth forests in the circumboreal zone: implications for conservation, restoration and management. Silva Fennica.

[12] Brais S, David P, Ouimet R (2000). Impacts of wild fire severity and salvage harvesting on the nutrient balance of jack pine and black spruce boreal stands. Forest Ecology and Management.

[13] Bond-Lamberty B, Gower ST, Wang C, Cyr P, Veldhuis H (2006). Nitrogen dynamics of a boreal black spruce wildfire chronosequence. Biogeochemistry.

[14] Yermakov Z, Rothstein DE (2006). Changes in soil carbon and nitrogen cycling along a 72-year wildfire chronosequence in Michigan jack pine forests. Oecologia.

[15] Smithwick EA, Turner MG, Mack MC, Chapin FS (2005). Postfire soil N cycling in northern conifer forests affected by severe, stand-replacing wildfires. Ecosystems.

[16] Palviainen M (2017). Nitrogen balance along a northern boreal forest fire chronosequence. PloS one.

[17] Schaphoff S, Reyer CP, Schepaschenko D, Gerten D, Shvidenko A (2016). Tamm Review: Observed and projected climate change impacts on Russia’s forests and its carbon balance. Forest Ecology and Management.

[18] Arneth, A. *et al*. Historical carbon dioxide emissions caused by land-use changes are possibly larger than assumed. *Nature Geoscience* (2017).

[19] Valdez-Hernández M, Sánchez O, Islebe GA, Snook LK, Negreros-Castillo P (2014). Recovery and early succession after experimental disturbance in a seasonally dry tropical forest in Mexico. Forest Ecology and Management.

[20] Walker LR, Wardle DA, Bardgett RD, Clarkson BD (2010). The use of chronosequences in studies of ecological succession and soil development. Journal of Ecology.

[21] Bond‐Lamberty B, Wang C, Gower ST (2004). Net primary production and net ecosystem production of a boreal black spruce wildfire chronosequence. Global Change Biology.

[22] Mack MC (2008). Recovery of aboveground plant biomass and productivity after fire in mesic and dry black spruce forests of interior Alaska. Ecosystems.

[23] Wirth C (1999). Above-ground biomass and structure of pristine Siberian Scots pine forests as controlled by competition and fire. Oecologia.

[24] Johnson EA, Miyanishi K (2008). Testing the assumptions of chronosequences in succession. Ecology Letters.

[25] Rogers BM, Soja AJ, Goulden ML, Randerson JT (2015). Influence of tree species on continental differences in boreal fires and climate feedbacks. Nature Geoscience.

[26] Harmon, M. E. In *Old-Growth Forests* 159–190 (Springer, 2009).

[27] Schulze E-D, Wirth C, Mollicone D, Ziegler W (2005). Succession after stand replacing disturbances by fire, wind throw, and insects in the dark Taiga of Central Siberia. Oecologia.

[28] Larjavaara M (2010). Maintenance cost, toppling risk and size of trees in a self-thinning stand. Journal of Theoretical Biology.

[29] Kajimoto, T., Osawa, A., Usoltsev, V. & Abaimov, A. In *Permafrost Ecosystems* 99–122 (Springer, 2010).

[30] Piha A, Kuuluvainen T, Lindberg H, Vanha-Majamaa I (2013). Can scar-based fire history reconstructions be biased? An experimental study in boreal Scots pine. Canadian Journal of Forest Research.

[31] Berg B, Dise N (2004). Calculating the long-term stable nitrogen sink in northern European forests. Acta Oecologica.

[32] Akselsson C, Westling O (2005). Regionalized nitrogen budgets in forest soils for different deposition and forestry scenarios in Sweden. Global Ecology and Biogeography.

[33] Kukavskaya EA (2012). Fire emissions estimates in Siberia: evaluation of uncertainties in area burned, land cover, and fuel consumption. Canadian Journal of Forest Research.

[34] MacDicken KG (2015). Global forest resources assessment 2015: What, why and how?. Forest Ecology and Management.

[35] Stocker, T. F. *et al*. (Cambridge University Press Cambridge, UK, and New York, 2014).

[36] Myneni R (2001). A large carbon sink in the woody biomass of northern forests. Proceedings of the National Academy of Sciences.

[37] Gauthier S, Bernier P, Kuuluvainen T, Shvidenko A, Schepaschenko D (2015). Boreal forest health and global change. Science.

[38] Gustafson EJ, Shvidenko AZ, Sturtevant BR, Scheller RM (2010). Predicting global change effects on forest biomass and composition in south‐central Siberia. Ecological Applications.

[39] Simard M, Lecomte N, Bergeron Y, Bernier PY, Paré D (2007). Forest productivity decline caused by successional paludification of boreal soils. Ecological Applications.

[40] Rolstad, J., Blanck, Y. l. & Storaunet, K. O. Fire history in a western Fennoscandian boreal forest as influenced by human land use and climate. *Ecological Monographs* (2017).

[41] Kuuluvainen T (2009). Forest management and biodiversity conservation based on natural ecosystem dynamics in northern Europe: the complexity challenge. AMBIO: A Journal of the Human Environment.

[42] Wardle DA (2012). Linking vegetation change, carbon sequestration and biodiversity: insights from island ecosystems in a long-term natural experiment. Journal of Ecology.

[43] Häkkinen M, Heikkinen J, Mäkipää R (2011). Soil carbon stock increases in the organic layer of boreal middle-aged stands. Biogeosciences.

[44] Sievänen R (2014). Carbon stock changes of forest land in Finland under different levels of wood use and climate change. Annals of forest science.

[45] Troeva, E. I., Isaev, A. P., Cherosov, M. & Karpov, N. *The Far North:: Plant Biodiversity and Ecology of Yakutia*. Vol. 3 (Springer Science & Business Media, 2010).

[46] Grieser J, Gommes R, Bernardi M (2006). New LocClim–the local climate estimator of FAO. Geophysical Research Abstracts.

[47] Anisimov O, Reneva S (2006). Permafrost and changing climate: the Russian perspective. AMBIO: A Journal of the Human Environment.

[48] Larjavaara M, Muller-Landau HC (2013). Measuring tree height: a quantitative comparison of two common field methods in a moist tropical forest. Methods in Ecology and Evolution.

[49] Larjavaara M, Muller-Landau HC (2010). Comparison of decay classification, knife test, and two penetrometers for estimating wood density of coarse woody debris. Canadian Journal of Forest Research-Revue Canadienne De Recherche Forestiere.

[50] Thomas SC, Martin AR (2012). Carbon content of tree tissues: a synthesis. Forests.

[51] Hytönen, J., Saarsalmi, A. & Rossi, P. Biomass production and nutrient uptake of short-rotation plantations (1995).

[52] Uri V, Tullus H, Lõhmus K (2003). Nutrient allocation, accumulation and above-ground biomass in grey alder and hybrid alder plantations. Silva Fennica.

[53] Brais S, Paré D, Lierman C (2006). Tree bole mineralization rates of four species of the Canadian eastern boreal forest: implications for nutrient dynamics following stand-replacing disturbances. Canadian Journal of Forest Research.

[54] Kuznetsova T, Lukjanova A, Mandre M, Lõhmus K (2011). Aboveground biomass and nutrient accumulation dynamics in young black alder, silver birch and Scots pine plantations on reclaimed oil shale mining areas in Estonia. Forest Ecology and Management.

[55] Palviainen M, Finér L (2012). Estimation of nutrient removals in stem-only and whole-tree harvesting of Scots pine, Norway spruce, and birch stands with generalized nutrient equations. European Journal of Forest Research.

[56] Harmon, M. E., Woodall, C. W., Fasth, B., Sexton, J. & Yatkov, M. Differences between standing and downed dead tree wood density reduction factors: a comparison across decay classes and tree species. (2011).

[57] Cunia T, Briggs R (1984). Forcing additivity of biomass tables: some empirical results. Canadian Journal of Forest Research.

[58] Repola J (2008). Biomass equations for birch in Finland. Silva Fennica.

[59] Repola J (2006). Models for vertical wood density of Scots pine, Norway spruce and birch stems, and their application to determine average wood density. Silva Fennica.

[60] Chave J (2009). Towards a worldwide wood economics spectrum. Ecology Letters.

[61] Mäkinen H, Hynynen J, Siitonen J, Sievaneni R (2006). Predicting the decomposition of Scots pine, Norway spruce, and birch stems in Finland. Ecological Applications.

[62] Krankina ON, Harmon ME, Griazkin AV (1999). Nutrient stores and dynamics of woody detritus in a boreal forest: modeling potential implications at the stand level. Canadian Journal of Forest Research.

[63] Palviainen M, Laiho R, Mäkinen H, Finer L (2008). Do decomposing Scots pine, Norway spruce, and silver birch stems retain nitrogen?. Canadian Journal of Forest Research.

