# Supplementary material for: Post-fire carbon and nitrogen accumulation and succession in Central Siberia
Source: Sci Rep. 2017 Oct 6;7:12776. doi: 10.1038/s41598-017-13039-2 (PMC5630608; doi:10.1038/s41598-017-13039-2)
Supplement: Supplementary file 1 — Supplementary Info File [file 41598_2017_13039_MOESM1_ESM.pdf]

Post-fire carbon and nitrogen accumulation and succession in Central Siberia

Markku Larjavaara, Frank Berninger, Marjo Palviainen, Anatoly Prokushkin, Tuomo Wallenius

1. Methods and results when taking legacy of earlier fires into account

It is likely that ecosystems are influenced significantly by earlier fires than the previous one. Because the previous fire is likely to have a stronger impact on ecosystem state than any of the earlier fires, understanding their impact is even more challenging.

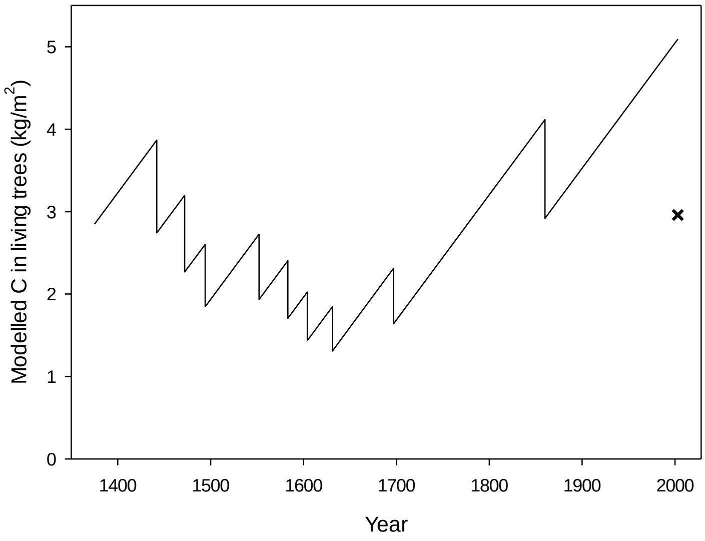

Supplementary Figure S1. Modelled carbon density trajectory in living trees for the plot 205 having the most dated fires. The very frequent fires with shorter interval than 77 years lower the carbon density in living trees during the first half of the simulation period while the opposite is true for the second half. The cross marks the measured carbon density in living trees.

We experimented a simple modelling approach to take all the dated fires into account in explaining C in living trees. We first assumed that the higher is the living tree biomass before the fire, the higher it is after it and that the proportion reduced in a fire is constant that we obtained based on the mean 77-old fire cycle, and the linear regression of the top right panel of Figure 2. The recovery of C in living trees starts at 2.85 kg/m^2, and increases annually 0.0152 kg/m^2. Therefore, according to this regression model the C density at the end of the 77-year succession is 4.02 kg/m^2 and the proportion remaining after fire was 0.709. We assumed this reduction for all fires. These assumptions can be used to compute C density trajectories as we show in Supplementary Figure S1.

With the assumption of 70.9% of the C accumulated in the living trees remaining the following rotation, the impact of the preceding rotation to the final C density in living trees is only 70.9% of the subsequent rotation. To explain C density in living trees a mean can be taken by weighting more the more recent fire cycles. This can be expressed mathematically:

$$w = \frac{\sum_{i=0}^{n}(f_i \cdot p_i^n)}{\sum_{i=0}^{n}(p_i^n)}$$

, where *w* is the "weighted mean time since fire", *n* is the rotation with 0 being the ongoing period since fire, 1 the fire interval before previous fire, *f* the fire interval or (or in the case of the ongoing period, time since fire) and *p* the proportion of C remaining after fire (70.9 %). The result of this approach can be seen as Supplementary Figure S2.

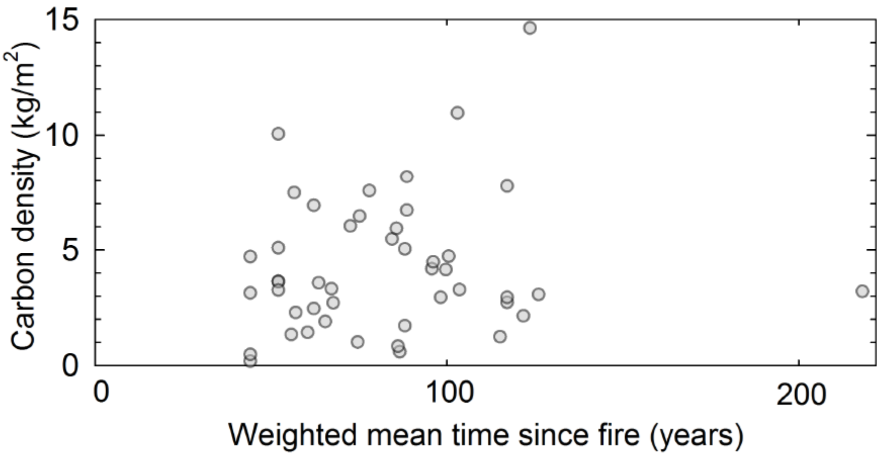

Supplementary Figure S2. C density of living trees explained with the weighted mean time since fire that takes into account not only the previous fire but all dated fires.

2. Photographs from the plots

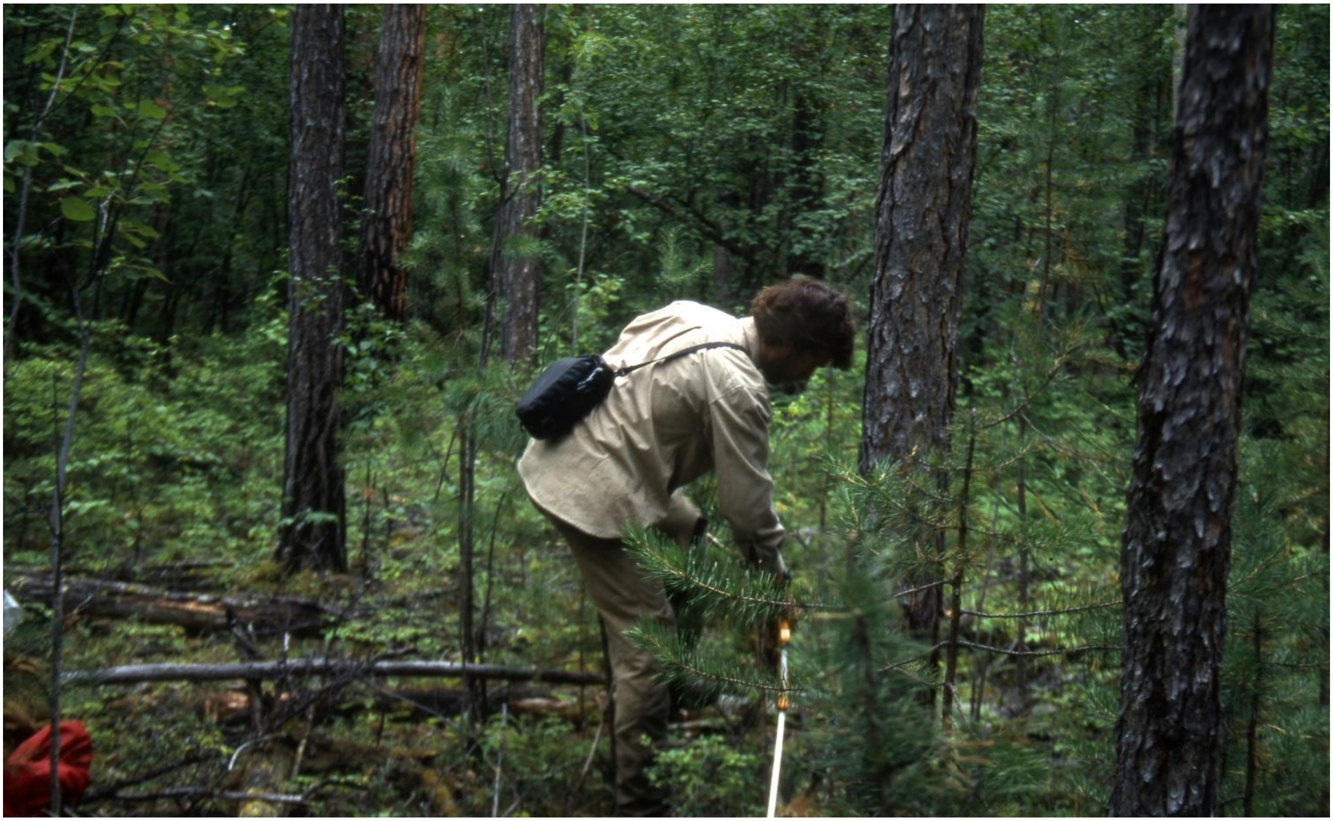

ML setting one of the plots of the southernmost hike.

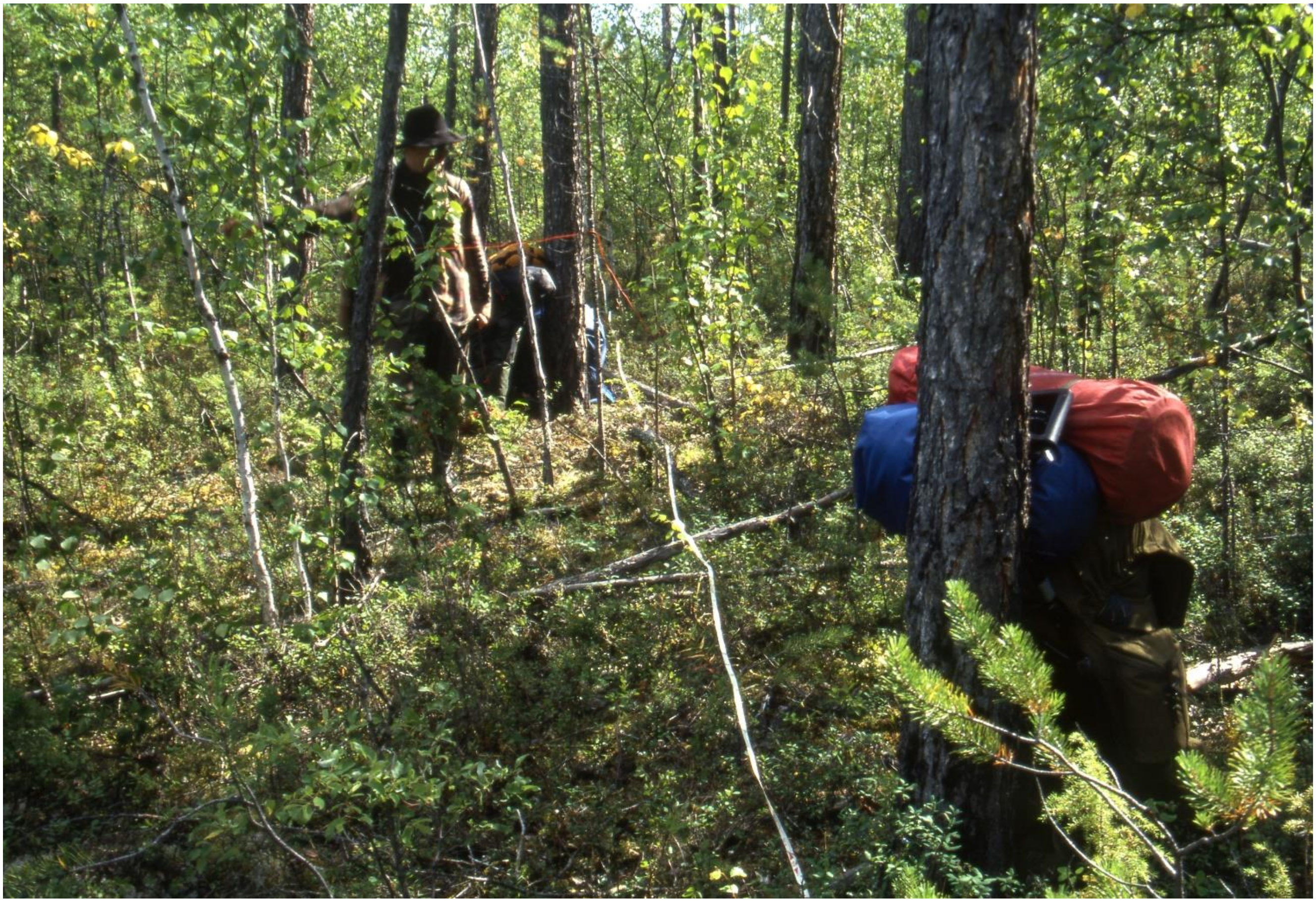

TW measuring small trees in another plot of the southernmost hike.

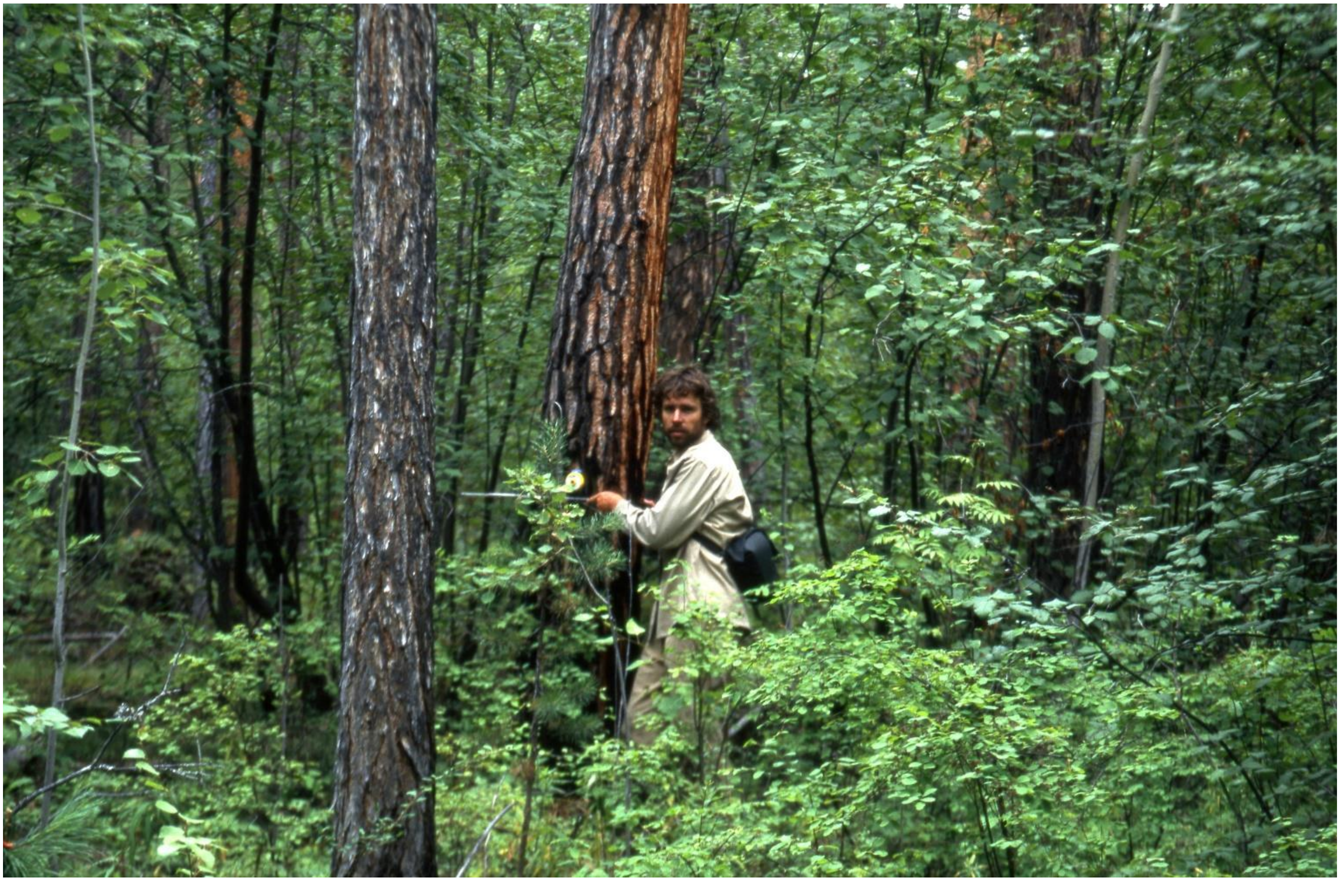

ML measuring the dbh of a large *Larix* tree in one of the plots of the southernmost hike.

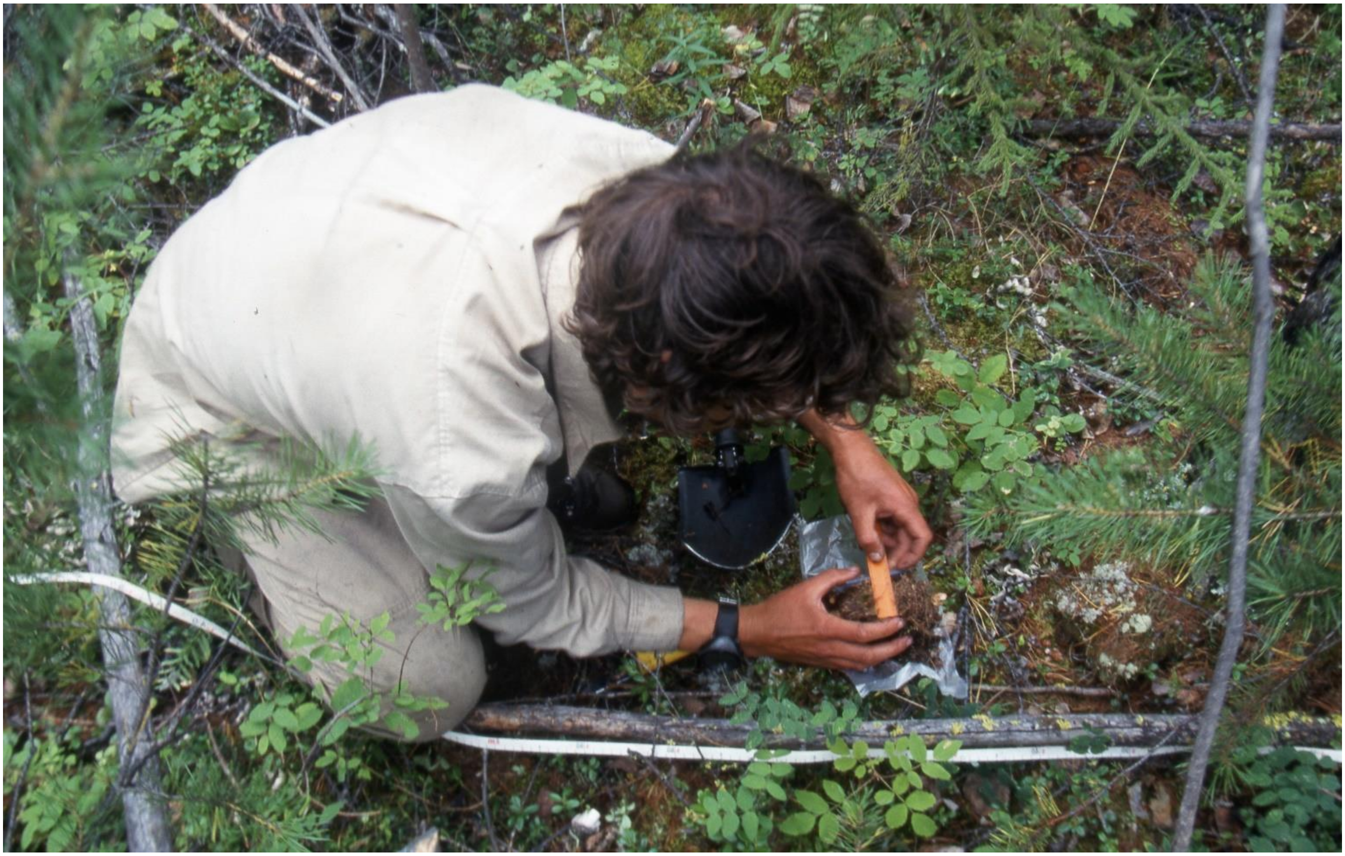

ML measuring the thickness of a soil sample in one of the plots of the southernmost hike.

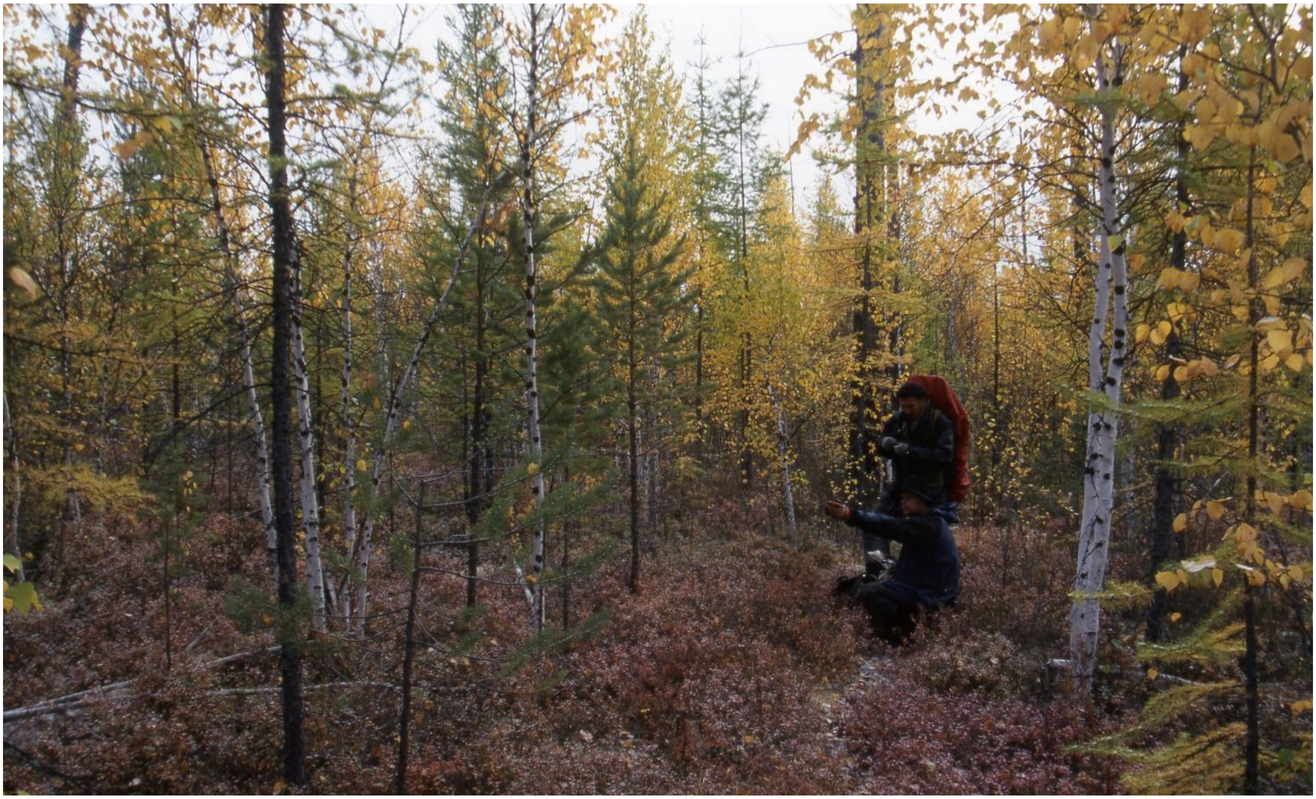

TW showing the direction of the central 20 m line of the plot to Aleksey Sadvordaev in one of the plots of the northernmost hike.

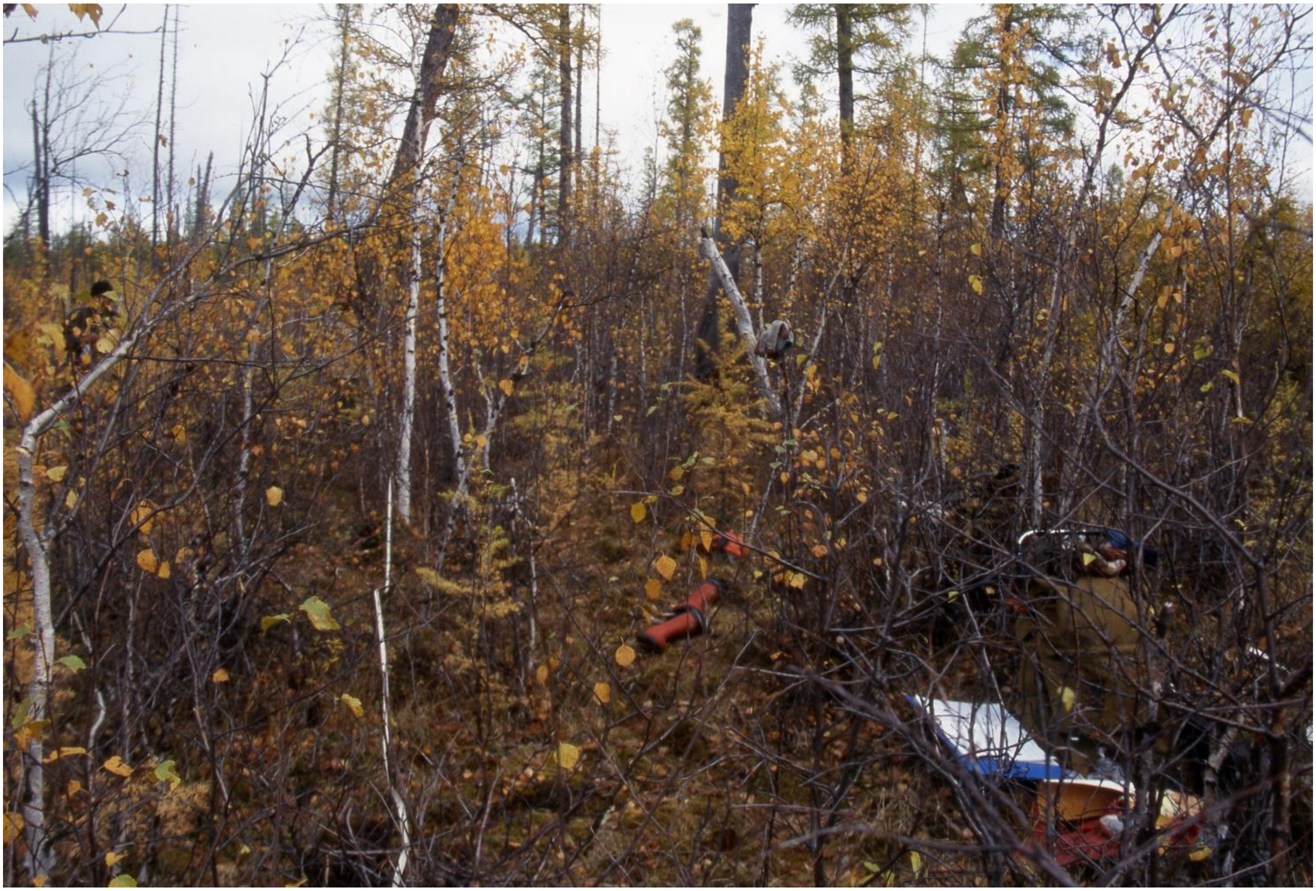

One of the plots of the northermmost hike.

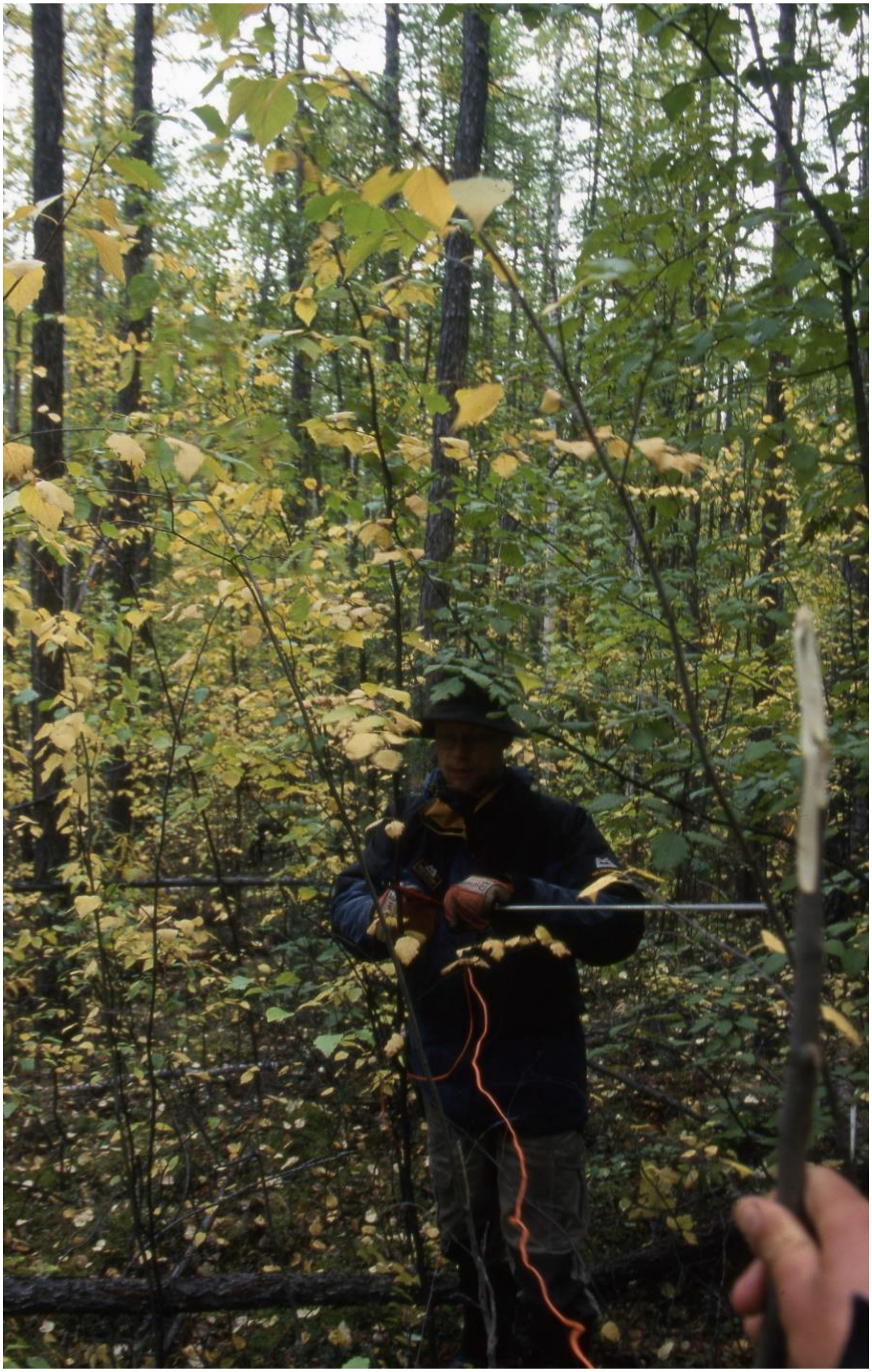

TW measuring diameter of a small tree and the photographer (ML) estimating its height with the help of a stick in one of the plots of the northernmost hike.

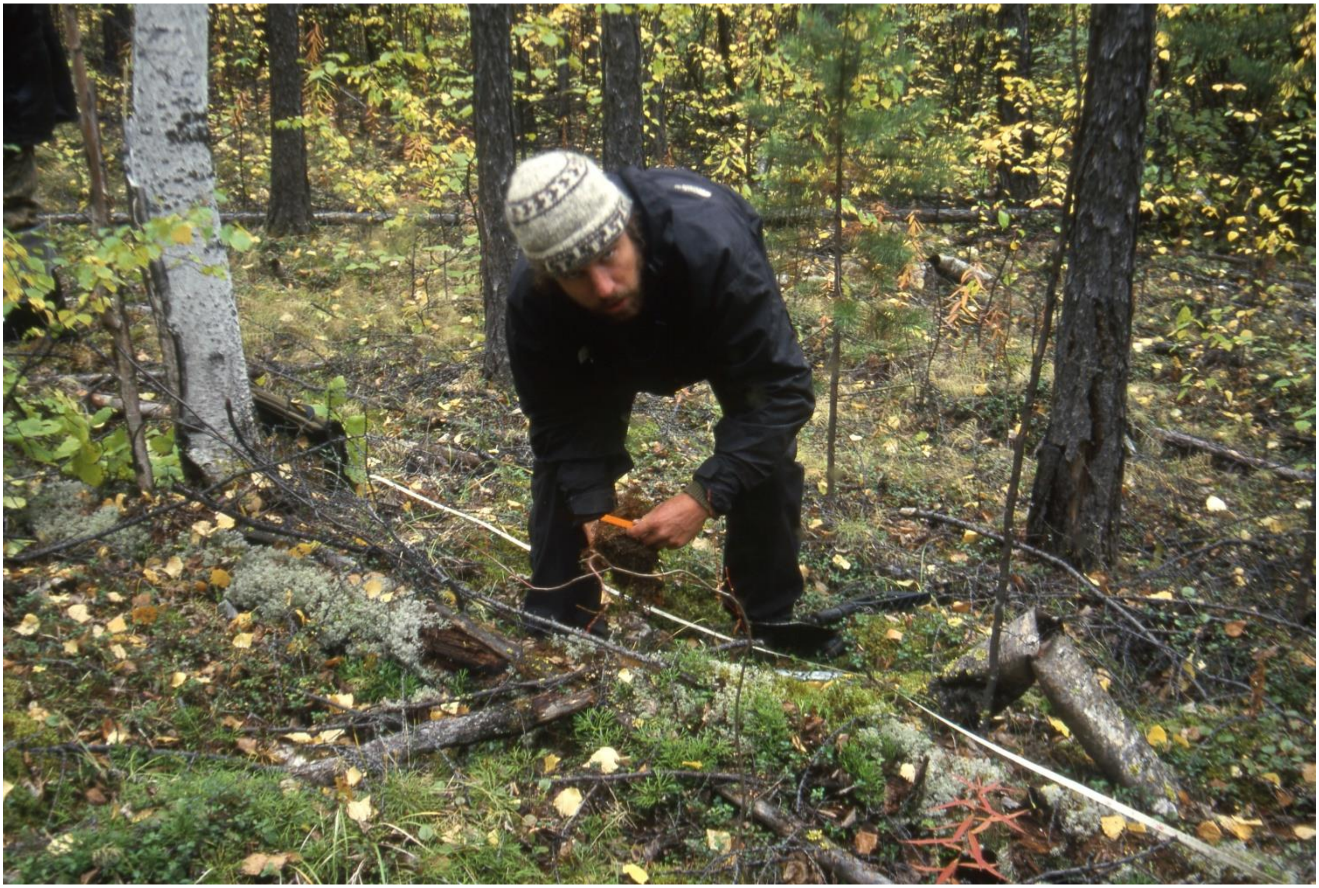

MLmeasuring the thickness of a soil sample in one of the plots of the northernmost hike.

3. Allometric equation on above-ground biomass for *Larix gmelinii*

We developed allometric biomass equations for *Larix gmelinii* based on plots in the vicinity of Tura settlement (64°19’ N, 100°15’E). We established the plots based on Russian inventory methods by including two hundred trees by varying the plot size. From these trees we selected sample trees representing each diameter class (with 1cm or 2cm steps depending on the number of trees) sample trees. These trees were felled and then we obtained the fresh mass by separately weighing the trunks and crown, including the branches and needles, in the field. We used a balance with 0.05 kg precision for trees that were over 25 kg and a balance with 0.01 kg precision for smaller trees. We then cut discs from the base of the trunk, at the dbh, at two thirds of the height and three fourths of the height and transported these in closed plastic bags to the laboratory for fresh mass weighting and subsequent drying. Similarly, we took samples of both thick (> 2 cm) and thin branches from the bottom, middle and top part of the crown. We then dried the discs and sampled branches at 105°C for 12 hours to obtain the dry mass. We obtained the biomass of the trees by assuming the same water content for the non-sampled parts of the felled trees as for the respective fractions that were sampled. Finally, we fitted a non-linear model with Statistica 6.0 relating above-ground biomass (AGB) to dbh and tree height (h):

$$AGB = 0.043691\, h\, (DBH)^{1.781}.$$
